# Usefulness of ascitic fluid lactoferrin levels in patients with liver cirrhosis

**DOI:** 10.1186/s12876-016-0546-9

**Published:** 2016-10-13

**Authors:** Sang Soo Lee, Hyun Ju Min, Ja Yun Choi, Hyun Chin Cho, Jin Joo Kim, Jae Min Lee, Hong Jun Kim, Chang Yoon Ha, Hyun Jin Kim, Tae Hyo Kim, Jin Hyun Kim, Ok-Jae Lee

**Affiliations:** 1 Department of Internal Medicine, Gyeongsang National University School of Medicine and Gyeongsang National University Hospital, 15, Jinju-daero 816 beon-gil, Jinju, Gyeongnam 52727 Republic of Korea; 2 Institute of Health Sciences, Gyeongsang National University, Jinju, Republic of Korea; 3 Biomedical Research Institute, Gyeongsang National University Hospital, Jinju, Republic of Korea; 4 Department of Internal Medicine, Gyeongsang National University School of Medicine and Gyeongsang National University Changwon Hospital, Jinju, Republic of Korea

**Keywords:** Lactoferrin, Ascites, Spontaneous bacterial peritonitis, Liver cirrhosis, Hepatocellular carcinoma

## Abstract

**Background:**

Although elevated levels of lactoferrin provide a biomarker for inflammatory bowel diseases and colorectal cancer, the clinical significance of these elevated levels in ascitic fluid of patients with ascites caused by liver cirrhosis is limited. The aims of our study were to investigate the usefulness of ascitic fluid lactoferrin levels for the diagnosis of spontaneous bacterial peritonitis (SBP) in patients with cirrhosis and to evaluate the association between lactoferrin levels and the development of hepatocellular carcinoma (HCC).

**Methods:**

A total of 102 patients with ascites caused by cirrhosis were consecutively enrolled into the study, from December 2008 to December 2011. Ascitic fluid lactoferrin levels were quantified using a human lactoferrin enzyme-linked immunosorbent assay kit.

**Results:**

The median ascitic fluid lactoferrin levels were significantly higher in patients with SBP than in those without SBP (112.7 ng/mL vs. 0.6 ng/mL; *p* < 0.001). The area under the receiver operator characteristic curve for the diagnosis of SBP was 0.898 (95 % confidence interval, 0.839–0.957, *p* < 0.001), with a sensitivity and specificity for a cut-off level of 51.4 ng/mL of 95.8 % and 74.4 %, respectively. Moreover, the incidence of HCC in the 78 patients without SBP was significantly higher in patients with high ascitic fluid lactoferrin levels (≥35 ng/mL) than in those with low ascitic fluid lactoferrin level (<35 ng/mL).

**Conclusions:**

Ascitic fluid lactoferrin level can be a useful diagnostic tool to identify SBP in patients with ascites caused by cirrhosis. Elevated ascitic fluid lactoferrin level in patients without SBP may be indicative of a developing hepatocellular carcinoma.

**Electronic supplementary material:**

The online version of this article (doi:10.1186/s12876-016-0546-9) contains supplementary material, which is available to authorized users.

## Background

Lactoferrin is a 78-kDa iron-binding protein in the transferrin family [1]. Lactoferrin is found in bovine milk, as well as in human breast milk [2]. This iron-binding protein is also present in mucosal secretions, including gastrointestinal fluids, saliva, tears, semen, vaginal fluids, nasal fluid, and bronchial mucosa [3, 4]. Lactoferrin is believed to have several relevant functions, that include anticancer, antibacterial, antiviral, antifungal, antiparasitic, anti-inflammatory, anti-oxidant and immune regulatory activities [5, 6]. During an infection or inflammatory condition, lactoferrin is expressed and secreted from polymorphonuclear cells (PMNs) and lactoferrin levels are elevated in the body. Lactoferrin levels have been shown to increase in the presence of infection or inflammatory condition [7]. Therefore, an elevated lactoferrin level may provide a promising and reliable biomarker for gastrointestinal disease [8]. Lactoferrin levels are elevated not only in patients with inflammatory bowel diseases, such as ulcerative colitis and Crohn’s disease, but also in patients with colorectal cancer [9–12]. Recently, several studies have provided evidence that increased systemic inflammation is associated with poor survival in various cancers [13]. In patients with hepatocellular carcinoma (HCC), these systemic inflammatory responses can be detected by routine laboratory tests, such as levels of C-reactive protein (CRP) and the neutrophil-lymphocyte ratio (NLR) [14–16]. However, the association between ascitic fluid lactoferrin levels and the development of HCC in patients with ascites caused by cirrhosis has not been investigated to date.

Ascites is one of the most common complications of advanced liver disease [17]. Spontaneous bacterial peritonitis (SBP) is a clinical syndrome in which ascitic fluid becomes infected in the absence of any apparent intra-abdominal source of peritonitis [18]. The diagnosis of SBP is based on a manual count of PMN cells in ascitic fluid, with counts ≥ 250 cells/mm^3^ indicative of SBP. However, this method of diagnosis is operator-dependent and, therefore, subject to human error. Moreover, lysis of the cells during transport to the laboratory can lead to false negative results.

Parsi et al. assessed the utility of ascitic fluid lactoferrin level for the diagnosis of SBP in patients with cirrhosis [19]. However, few studies have evaluated the findings of Parsi et al. to clarify the clinical usefulness of ascitic fluid lactoferrin level for the diagnosis of SBP in patients with liver cirrhosis. Therefore, the aims of our study were to investigate the usefulness of ascitic fluid lactoferrin levels for the diagnosis of SBP in patients with ascites caused by cirrhosis and to evaluate the association between lactoferrin levels and the development of HCC.

## Methods

### Study population

A prospective cohort study group was formed by consecutive enrollment of 182 patients with ascites caused by cirrhosis, from December 2008 to December 2011, at Gyeongsang National University Hospital in South Korea. The inclusion criteria were: (1) known diagnosis of cirrhosis, (2) age ≥20 years, and (3) presence of grade 2 or 3 ascites, based on the definitions of the International Ascites Club [20]. The exclusion criteria were as follows: (1) presence of HCC (*n* = 75) and (2) other causes of neutrocytic ascites, including peritoneal carcinomatosis, tuberculosis, pancreatitis, appendicitis, and hemorrhagic ascites (*n* = 5). A total of 102 patients met our inclusion and exclusion criteria and formed our study group. The Institutional Review Board of Gyeongsang National University Hospital reviewed and approved this study and all patients provided informed consent.

### Definitions

Liver cirrhosis was defined by the presence of portal hypertension manifested as splenomegaly, varices, ascites, or hepatic encephalopathy, with compatible findings on diagnostic imaging, in combination with thrombocytopenia (<100,000/μl). The diagnosis of HCC was based on histology or typical radiological findings of hepatic nodules on arterial enhancement and venous wash-out, contrast-enhanced computed tomography (CT) or magnetic resonance (MR) imaging [21]. The diagnosis of SBP was based on a PMN count ≥250 cells/mm^3^ in ascitic fluid, with or without a positive ascitic fluid or blood culture. Ascitic fluid samples were obtained from patients with grade 2 or 3 ascites for cell count, culture and determination of lactoferrin levels.

### Data collection

Clinical data including age, sex, alcohol consumption, the presence of hypertension and/or diabetes, the etiology of cirrhosis, and prior SBP history were obtained. At enrollment, laboratory tests were performed for anti-hepatitis C virus (HCV), hepatitis B virus surface antigen (HBsAg), anti-hepatitis B virus surface antibody (anti-HBs), white blood cell (WBC) count, hemoglobin level, platelet count, prothrombin time- international normalized ratio (PT-INR), total bilirubin, aspartate aminotransferase (AST), alanine aminotransferase (ALT), gamma glutamyl transpeptidase, serum albumin, creatinine, CRP, and ascitic fluid analysis, including WBC count, PMN count, and albumin levels. In addition, the Child-Pugh score was determined. The ascitic fluid samples (50 mL) were frozen at −70 °C immediately after collection until analyzed. The lactoferrin level in ascitic fluid was quantified using a human lactoferrin enzyme-linked immunosorbent assay kit according to the manufacturer’s instructions (Bethyl Laboratories, Inc., Tokyo, Japan). This kit, designed as a sandwich ELISA, captures human lactoferrin present in samples by anti-lactoferrin antibody that has been pre-adsorbed on the surface of polystyrene microtiter wells. The lactoferrin levels were quantified by interpolating their absorbance (at 450 nm) from the standard curve.

### Patient follow-up

All patients were closely monitored for clinical and biochemical status, with imaging examinations, by ultrasound or CT, completed every 3 to 12 months. The cumulative death rate was measured from the date of enrollment until the date of death, the last follow-up, or to the study end date of December 31, 2015. Assessment of survival included data obtained by telephone interview with patients or one of their family members. To calculate the true incidence of new HCC cases based on ascitic fluid lactoferrin levels, patients with less than 6 months of follow-up, patients diagnosed with HCC within 6 months of enrollment or patients with SBP were excluded. Based on these exclusion criteria, 24 patients removed from the calculation of the true incidence of new HCC cases due to development of SBP within 6 months of enrollment.

### Statistical analysis

Data were presented as median (interquartile range) for continuous variables, and as frequency and percentage for categorical variables. Differences between patients with and without SBP were evaluated using the chi-squared test or 2-tailed Fischer’s exact test for categorical variables, and the Mann–Whitney test was used for continuous variables. Bivariate correlations were performed using Spearman’s Rank Correlation to evaluate the correlation of lactoferrin levels to all other measured study variables. Receiver operator characteristic (ROC) analysis, area under the curve (AUC) and 95 % confidence interval (CI) of the AUC were used to identify the optimal cutoff value of lactoferrin level for the diagnosis of SBP. A *p*-value <0.05 indicated statistical significance. Statistical analyses were performed using PASW software (Version 18, SPSS Inc., Chicago, IL, USA).

## Results

### Patients’ characteristics

Demographic and clinical characteristics of the 102 patients forming our study group are summarized in Table 1. Liver cirrhosis was caused by alcohol in 64.7 %, HBV in 17.6 %, HCV in 11.8 %, and HBV + HCV in 1.0 %, with the remaining 4.9 % of patients having a diagnosis liver of disease from ‘other’ causes. SBP was diagnosed in 24 patients (22.9 %).

**Table 1:** Baseline demographic and clinical characteristics according to spontaneous bacterial peritonitis

	Total (*n* = 102)	No SBP (*n* = 78)	SBP (*n* = 24)	*p*-value
Age, years	54.5 (45.8–62.8)	53.0 (45.8–62.0)	55.5 (45.8–68.5)	0.376
Gender, male	73 (71.6 %)	59 (75.6 %)	14 (58.3 %)	0.123
Etiology				0.983
Alcohol	66 (64.7 %)	50 (64.1 %)	16 (66.7 %)	
HBV	18 (17.6 %)	14 (17.9 %)	4 (16.7 %)	
HCV	12 (11.8 %)	9 (11.5 %)	3 (12.5 %)	
HBV + HCV	1 (1.0 %)	1 (1.3 %)	0 (0 %)	
Others	5 (4.9 %)	4 (5.1 %)	1 (4.2 %)	
Child-Pugh score	11 (9–12)	11 (9–12)	11 (10–12)	0.089
Prior SBP	19 (18.6 %)	12 (15.4 %)	7 (29.2 %)	0.143
Ascitic fluid level				
Lactoferrin (ng/ml)	21.7 (0–100.1)	0.6 (0–54.5)	112.7 (91.5–139.8)	<0.001
WBC (cells/ml)	151 (60–416)	100.5 (48.8–181.3)	1158 (538–5651)	<0.001
PMN (cells/ml)	11.5 (4.0–98.0)	6.5 (3.0–16.3)	5695 (3660–9668)	<0.001
Serum level				
WBC (×1000/μl)	6.2 (4.7–9.8)	5.9 (5.0–8.9)	7.6 (5.5–11.8)	0.037
PMN (×1000/μl)	3.9 (2.4–6.6)	3.7 (2.3–5.6)	5.7 (3.7–9.7)	0.006
Hemoglobin (g/dL)	9.3 (8.5–10.8)	9.3 (8.4–10.7)	9.2 (8.6–10.9)	0.862
Platelet (×1000/μl)	82.0 (50.0–127.8)	90.5 (53.0–135.3)	60.0 (47.3–105.5)	0.053
Albumin (g/dL)	2.5 (2.3–2.8)	2.6 (2.3–2.8)	2.5 (2.3–2.6)	0.190
Bilirubin (g/dL)	3.8 (2.0–7.8)	3.6 (1.9–7.7)	4.4 (2.3–12.7)	0.289
PT-INR	1.61 (1.35–2.23)	1.58 (1.33–2.07)	1.97 (1.49–2.39)	0.069
CRP (mg/L)	13.9 (6.7–21.9)	11.0 (5.2–16.4)	30.7 (18.7–68.1)	<0.001

Data are presented as the median (interquartile range) for continuous data and percentages for categorical data

*SBP* spontaneous bacterial peritonitis, *HBV* hepatitis B virus, *HCV* hepatitis C virus, *WBC* White blood cells, *PMN* Polymorphonuclear cell, *PT-INR* prothrombin time-international normalized ratio, *CRP* C-reactive protein

Median age, sex, disease etiology, Child-Pugh score, and incidence of prior SBP were comparable among patients with and without SBP. In laboratory results, median ascitic fluid lactoferrin level, ascitic WBC count, ascitic PMN count, serum WBC count, serum PMN count, and CRP level were significantly higher in patients with SBP than in those without SBP. The median ascitic fluid lactoferrin level was 0.6 in patients without SBP and 112.7 in patients with SBP (*p* <0.001).

### Correlation of ascitic fluid lactoferrin level with laboratory parameters

The correlations between ascitic fluid lactoferrin levels with laboratory parameters are summarized in Table 2. In all patients, ascitic fluid lactoferrin levels correlated with ascitic WBC count (*r* = 0.529, *p* <0.001), ascitic PMN count (*r* = 0.633, *p* < 0.001), serum PMN level (*r* = 0.200, *p* = 0.044), serum platelet count (*r* = −0.253, *p* = 0.018), serum CRP level (*r* = 0.355, *p* < 0.001), serum PT-INR (*r* = 0.232, *p* = 0.019), and the Child-Pugh score (*r* = 0.248, *p* = 0.012). In ascitic fluid or blood cultures, 13 of the 24 SBP patients (54.2 %) showed positive culture results for *Escherichia coli* (5, 20.8 %), *Klebsiella pneumoniae* (4, 16.7 %), *Streptococcus species* (2, 8.3 %), *Candida albicans* (1, 4.2 %), and *Clostridium Perfringens* (1, 4.2 %). The distribution of positive findings in patients with SBP is summarized in Table 3. In the 24 patients with SBP, there was no significant difference in ascitic fluid lactoferrin level between culture positive SBP and culture negative SBP (median 126.3 ng/ml vs. 104.0 ng/ml, *p* = 0.122).

**Table 2:** Correlation of ascitic lactoferrin level with clinical and laboratory variables in all patients (*n* = 102)

Variables	*r*	*P* value
Age (years)	0.082	0.415
WBC count in ascitic fluid (cells/mL)	0.529	<0.001
PMN count in ascitic fluid (cells/mL)	0.633	<0.001
Serum WBC count (cells/mL)	0.110	0.273
Serum PMN count (cells/mL)	0.200	0.044
Serum hemoglobin level (g/dL)	0.093	0.351
Serum platelet level (×1000/μl)	−0.253	0.018
Serum CRP	0.355	<0.001
Serum bilirubin level (g/dL)	0.132	0.187
Serum PT-INR	0.232	0.019
Serum albumin level (g/dL)	−0.023	0.820
Child-Pugh score	0.248	0.012

*WBC* White blood cells, *PMN* Polymorphonuclear cell, *CRP* C-reactive protein, *PT-INR* prothrombin time-international normalized ratio

**Table 3:** Causative microorganisms of spontaneous bacterial peritonitis (*n* = 24)

Organism	Number (%)
*Escherichia coli*	5 (20.8 %)
*Klebsiella pneumoniae*	4 (16.7 %)
*Streptococcus species*	2 (8.3 %)
*Candida albicans*	1 (4.2 %)
*Clostridium Perfringens*	1 (4.2 %)
No growth	11 (45.8 %)

Data are presented as number (%)

### Usefulness of ascitic fluid lactoferrin levels for the diagnosis of SBP

**Fig. 1:**
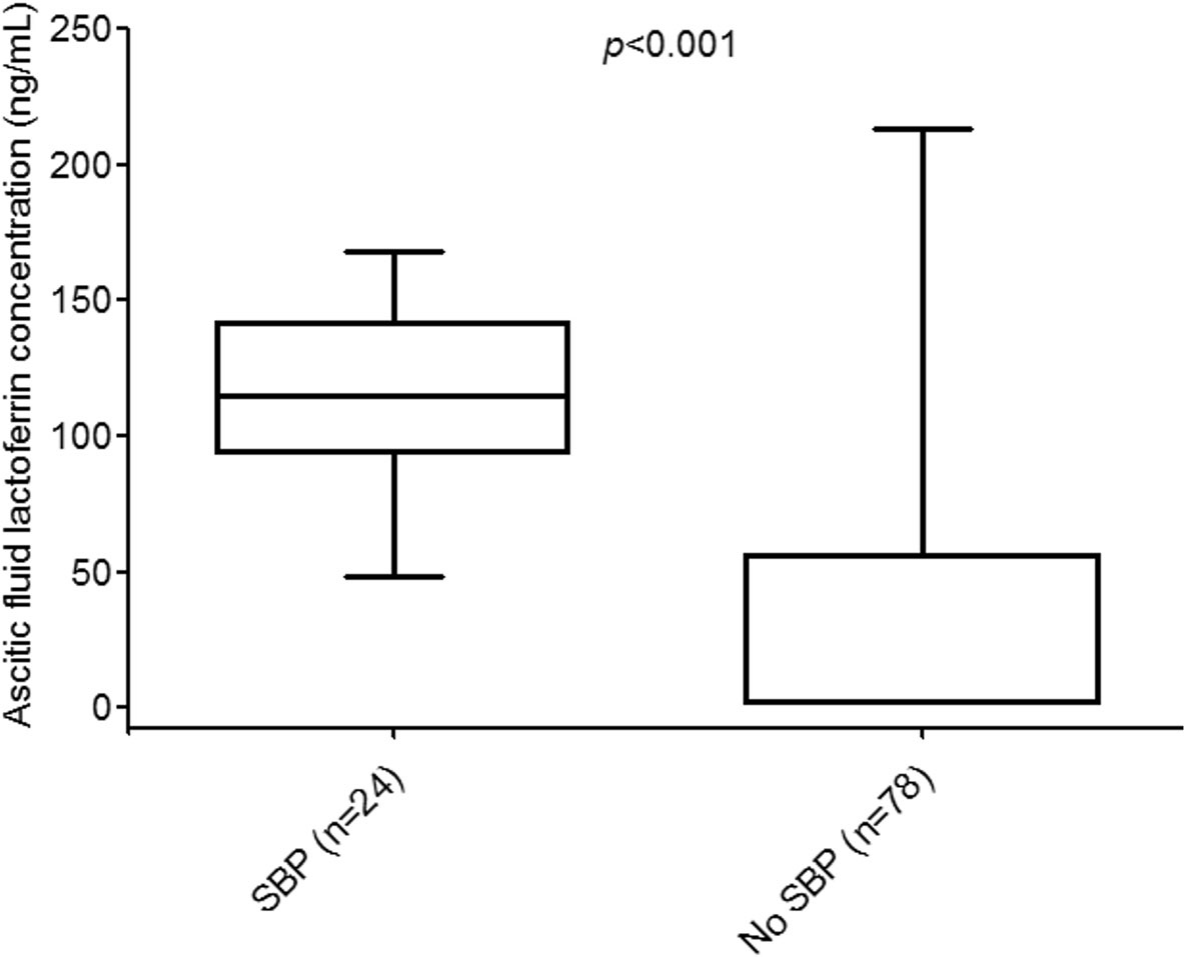
Ascitic fluid lactoferrin levels in patients with and without spontaneous bacterial peritonitis; SBP, spontaneous bacterial peritonitis

**Fig. 2:**
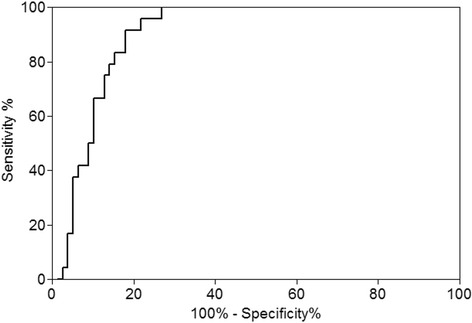
Receiver operating characteristic (ROC) curve of ascitic fluid lactoferrin levels for the diagnosis of spontaneous bacterial peritonitis (SBP) in patients with cirrhosis (*n* = 102); the area under the curve is 0.898, with a 95 % confidence interval of 0.839 to 0.957

The median ascitic fluid lactoferrin level in patients with SBP group was significantly higher than the level in patients without SBP (112.7 ng/mL vs. 0.6 ng/mL, *p *< 0.001; Fig. 1). Results of the ROC analysis are shown in Fig. 2. The area under the ROC curve for the diagnosis of SBP in the 102 patients with ascites caused by cirrhosis was 0.898 (95 % CI, 0.839–0.957, *p* < 0.001). The sensitivity and specificity for different cut-off levels of ascitic fluid lactoferrin for the diagnosis of SBP in this patient group are shown in Table 4. At the cut-off level of 51.4 ng/mL, the sensitivity and specificity of the test were 95.8 % and 74.4 %, respectively. At the cut-off level of 63.0 ng/mL, the sensitivity and specificity of the test were 91.7 % and 78.1 %, respectively.

**Table 4:** Diagnostic accuracy of ascitic fluid lactoferrin at the different cut-off levels for detection of spontaneous bacterial peritonitis in patients with cirrhosis (*n* = 102)

Ascites lactoferrin cut-off level (ng/mL)	Sensitivity (%)	Specificity (%)
43.0	100	72.1
51.4	95.8	74.4
63.0	91.7	78.1
76.3	83.3	82.1
95.9	75.0	87.2

Data are presented as number (%)

### Incidence of hepatocellular carcinoma

**Fig. 3:**
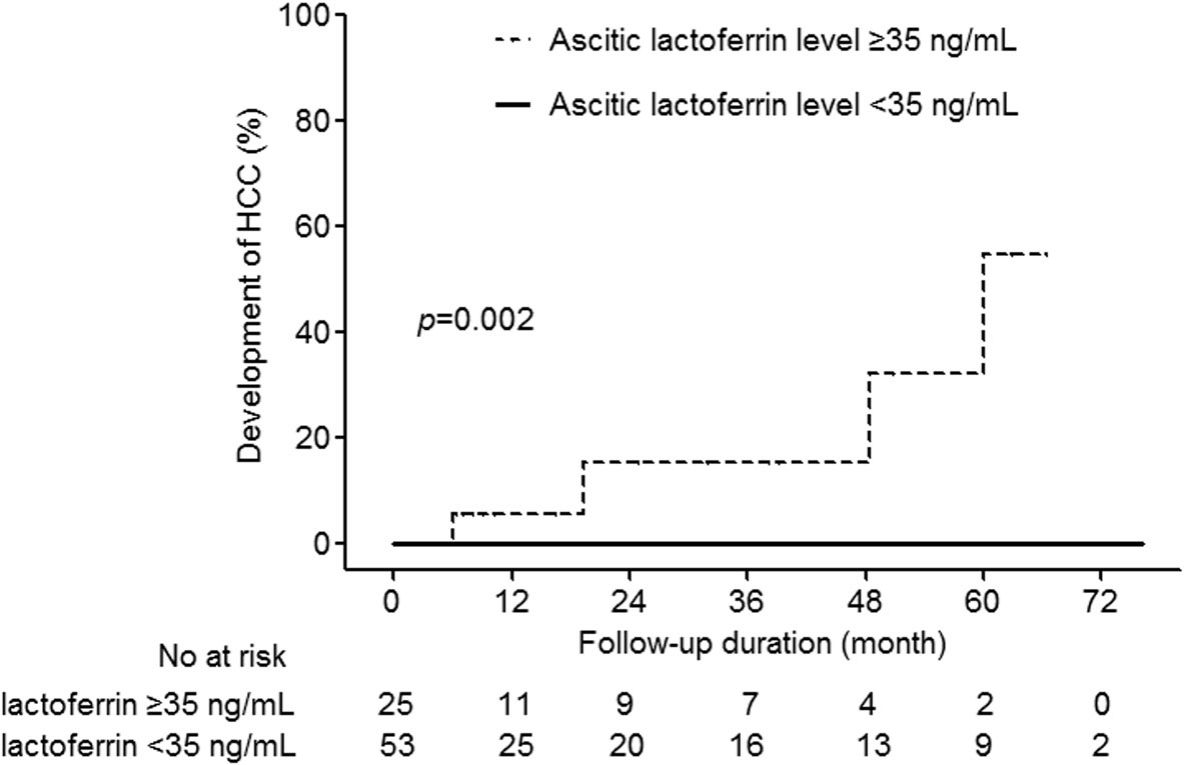
Cumulative incidence of hepatocellular carcinoma in patients with ascites caused by cirrhosis based on ascitic fluid lactoferrin level

We assessed the incidence of HCC development in the patients without SBP based on ascitic fluid lactoferrin levels. Of the 78 patients without SBP, 4 patients developed HCC during the study period. The cumulative incidence of HCC at 5 years was 17.9 % and the estimated yearly incidence of HCC development was 3.6 % in the first 5 years from the time of enrollment (Fig. 3). The cumulative incidence of HCC was significantly higher in patients with ascitic fluid lactoferrin levels ≥35 ng/mL than in those with ascitic fluid lactoferrin levels <35 ng/L (log rank test, *p* < 0.001).

## Discussion

Outcomes of our study provide evidence of the clinical usefulness of ascitic fluid lactoferrin levels in patients with cirrhosis to differentiate those with and without SBP. The area under the ROC for the diagnosis of SBP in the 102 patients with ascites caused by cirrhosis was 0.898 (95 % CI, 0.839–0.957, *p* < 0.001). The sensitivity and specificity of the ascitic fluid lactoferrin assay for the diagnosis of SBP in patients with ascites caused by cirrhosis were 95.8 % and 74.4 %, respectively, using a cut-off fluid level of 51.4 ng/mL. Moreover, the incidence of HCC development in patients without SBP was significantly higher for patients with high ascitic fluid lactoferrin levels, defined as a level ≥35 ng/mL.

Lactoferrin is released from PMNs during an infection or an inflammatory condition [7]. In the 102 patients with ascites caused by cirrhosis, lactoferrin levels in the ascitic fluid were significantly correlated with ascitic WBC count, ascitic PMN count, serum PMN count, serum platelet level, serum CRP, serum PT-INR, and the Child-Pugh score. Especially, high ascitic fluid lactoferrin levels were significantly correlated to inflammatory markers, including WBC, PMN, and CRP levels. It is important to note that the correlation of lactoferrin levels and inflammatory markers in blood samples and ascitic fluid could be influenced by lysis of PMN cells during transport to the laboratory, which could lead to a false negative result. Moreover, manual measurement of the ascitic fluid and PMN count is operator dependent, which makes quality control difficult. Commercially available kits for the measurement ascitic fluid lactoferrin could be used in a future development of a qualitative bedside assay. Moreover, lactoferrin is very stable and resistant to degradation at room temperature over an extended period and, therefore, a bedside assay would be feasible in making lactoferrin an important marker for SBP.

Parsi et al. assessed the utility of ascitic fluid lactoferrin level for the diagnosis of SBP in patients with cirrhosis [19] as a way of eliminating the risk for false negative results and diagnostic error associated with a manual count of ascitic fluid PMN cells. We confirm Parsi et al.’s conclusion regarding the clinical usefulness of ascitic fluid lactoferrin level as a biomarker for SBP in patients with ascites. However, our cut-off ascitic fluid lactoferrin level for the diagnosis of SBP was lower than the level identified by Parsi et al. A study by Ali et al. clarified the clinical usefulness of ascitic fluid lactoferrin as a biomarker for SBP [22]. The mean ascitic fluid lactoferrin levels was significantly higher in SBP patients (180.8 ng/ml) compared with patients without SBP (42.2 ng/ml, *P* = 0.001), with an ascitic lactoferrin level of 88 ng/ml identified as a cut-off on ROC analysis to distinguish patients ‘with’ and ‘without’ SBP. This cut-off of 88 ng/ml to identify SBP was lower than the cut-off ascitic lactoferrin level identified by Parsi et al., but higher than the cut-off level in our study. This difference in the cut-off of ascitic fluid lactoferrin level may be explained by the small sample sizes for SBP patients in both studies and, possibly, by differences in the etiology of cirrhosis. Thus, further multicenter studies are required to identify an optimal cut-off ascitic lactoferrin level for the diagnosis of SBP.

Recently, several studies provided evidence that increased systemic inflammation is associated with poor survival in various cancers [13]. In patients with HCC, these systemic inflammatory responses can be detected by routine laboratory tests, such as CRP level and the NLR [14–16]. In an immunohistochemistry study of liver biopsies in patients with viral and cryptogenic liver disease [23], lactoferrin was detected in 75 % of patients with chronic hepatitis. In HCV specimens, lactoferrin levels were found to increase with disease progression, suggesting that lactoferrin may play a role in modulating chronic liver inflammation [24]. However, there has been no report on the association between ascitic fluid lactoferrin levels and the development of HCC in patients with ascites caused by cirrhosis. Therefore, we hypothesized that ascitic fluid lactoferrin levels in patients with local inflammatory ascites would be higher than those in patients without local inflammatory ascites. The presence of ascitic fluid lactoferrin is proportional to the reflux of neutrophil, and local inflammation can provide a related marker for the development of HCC. In our study, we found that ascitic fluid lactoferrin level in patients without SBP might be related to the development of HCC. HCC free survival was 100 % and 45.2 % in patients with ascitic fluid lactoferrin levels <35 ng/L and ≥35 ng/mL, respectively. Thus, patients with ascitic fluid lactoferrin levels ≥35 ng/mL require more intensive surveillance for the early detection of HCC.

To our knowledge, this is the first prospective study to investigate the usefulness of lactoferrin levels in ascitic fluid for the diagnosis of SBP in patients with ascites due to cirrhosis in Korea. However, the limitations of our study need to be acknowledged in the interpretation and application of outcomes. Foremost, this is a single center prospective study with a relatively small study group, in which only 4 patients developed HCC during the study period. However, the ascitic fluid lactoferrin levels in these 4 patients were very high at 38.7 ng/mL, 60.5 ng/mL, 99.6 ng/mL, and 128.3 ng/mL. Therefore, further studies with larger study group that include patients with ascites caused by cirrhosis and HCC are needed. We also need to consider that the 102 patients with ascites caused by cirrhosis did not present with new onset ascites. Moreover, patients with ascites presented with different Child-Pugh scores, which could have introduced biases in our analysis of prognosis.

## Conclusion

Ascitic fluid lactoferrin level can be a useful indicator of SBP in patients with cirrhosis. Elevated ascitic fluid lactoferrin level in patients without SBP appears to be a promising predictor of HCC development. Therefore, larger multicenter studies are required to elucidate the usefulness of lactoferrin in ascitic fluid.

## Additional file

Additional file 1: The raw data of this study. All study materials and data are available. (XLS 74 kb)

## Abbreviations

ALT: Alanine aminotransferase
Anti-HBs: Anti-hepatitis B virus surface antibody
AST: Aspartate aminotransferase
AUC: Area under the curve
CI: Confidence interval
CRP: C-reactive protein
CT: Computed tomography
HBsAg: Hepatitis B virus surface antigen
HCC: Hepatocellular carcinoma
HCV: Hepatitis C virus
MR: Magnetic resonance
NLR: Neutrophil-lymphocyte ratio
PMN: Polymorphonuclear
PT-INR: Prothrombin time- international normalized ratio
ROC: Receiver operator characteristic
SBP: Spontaneous bacterial peritonitis
WBC: White blood cell
